# A Pragmatic Approach to Acute Cardiorenal Syndrome: Diagnostic Strategies and Targeted Therapies to Overcome Diuretic Resistance

**DOI:** 10.3390/jcm14092996

**Published:** 2025-04-26

**Authors:** Patrick Tran, Laith Khweir, Michael Kuehl, Mithilesh Joshi, Krishna Appunu, Waqar Ayub, Prithwish Banerjee

**Affiliations:** 1 Department of Cardiology and Renal Medicine, University Hospitals Coventry & Warwickshire, Clifford Bridge Road, Coventry CV2 2DX, UK; laith.khweir@uhcw.nhs.uk (L.K.); waqar.ayub@uhcw.nhs.uk (W.A.); prithwish.banerjee@uhcw.nhs.uk (P.B.); 2 Centre for Health and Life Sciences, Coventry University, Priority St., Coventry CV1 5FB, UK; 3 Medical School Building, University of Warwick, Coventry CV4 7AL, UK

**Keywords:** cardiorenal syndrome, diuretic resistance, ultrafiltration, ultrasound, inotropes

## Abstract

Cardiorenal syndrome (CRS) is a challenging condition characterised by interdependent dysfunction of the heart and kidneys. Despite advancements in understanding its pathophysiology, clinical management remains complex due to overlapping mechanisms and high rates of diuretic resistance. Relevant literature was identified through a comprehensive narrative review of PubMed, Embase, and Cochrane Library databases, focusing on pivotal trials relating to CRS from 2005 to 2024. This review aims to provide a pragmatic, evidence-based approach to acute CRS management by addressing common misconceptions, outlining diagnostic strategies, and proposing a structured algorithm to manage diuretic resistance. We discuss the role of thoracic and venous excess ultrasound (VeXUS) in providing reliable measures of systemic congestion, natriuresis-guided sequential nephron blockade, and more targeted therapies, including ultrafiltration in refractory cases. In addition, we explore emerging trials that target renal hypoperfusion and venous congestion in CRS. Designed for a broad audience, including general physicians, cardiologists, and nephrologists, this review integrates clinical evidence with practical guidance to support effective and timely decision-making in the care of patients with CRS.

## 1. Introduction

Cardiorenal syndrome (CRS) represents a delicate bidirectional balance between the heart and kidneys, where dysfunction in one can precipitate failure in the other. This intricate relationship is rooted in the shared haemodynamic, neurohormonal, and inflammatory pathways that govern both systems [1]. In heart failure (HF), reduced cardiac output (CO) and elevated venous pressures compromise renal perfusion, while neurohormonal activation exacerbates sodium and water retention, creating a vicious cycle that complicates treatment strategies [2]. Additionally, inflammatory mediators such as interleukin-6, tumour necrosis factor, and endothelin-1 exacerbate CRS by promoting endothelial dysfunction, oxidative stress, and tissue injury in both the heart and kidneys, amplifying the cycle of organ dysfunction [3]. A detailed review of the pathophysiology of CRS can be found elsewhere [1,3,4]. Although the classification of CRS by Ronco et al. (2008) [4] provides a conceptual framework (Table 1), its clinical utility is limited due to the challenges of identifying the primary driver of dysfunction in practice, compounded by the fact that >50% of acute and chronic HF patients have underlying chronic kidney disease (CKD) [4,5]. Notably, research has revealed that, for every 10 mL/min decrease in estimated glomerular filtration rate (eGFR), there is a 7% increase in mortality risk among patients with chronic HF [6]. This paper provides a pragmatic approach to CRS, addressing common misconceptions surrounding CRS and presenting evidence-based strategies for diagnosing congestion and managing diuretic resistance.

## 2. Common Misconceptions in Cardiorenal Syndrome

### 2.1. Arterial Underfilling Versus Venous Congestion

Low CO does not fully explain the cause of type 1 CRS. The kidneys are intrinsically high-flow, low-resistance organs that rely on both 20–25% of CO for adequate perfusion and low venous pressure to sustain the passive pressure gradient across the glomerular capillaries for effective filtration [2,7]. With low CO, renal blood perfusion falls as mean arterial pressure decreases, often below the renal autoregulatory threshold of around 65–80 mmHg, leading to diminished GFR [7]. This is often more pronounced in cardiogenic shock, where CO falls severely (i.e., cardiac index < 2.2 L/min/m^2^) to precipitate ischaemic ATN [5]. In terms of venous congestion, elevated central venous pressures transmit retrogradely into the renal veins, increasing interstitial hydrostatic pressure. This diminishes the arterial-venous pressure gradient, which ultimately reduces the GFR and potentially compresses the renal tubules within the encapsulated kidneys. Additionally, sympathetic-mediated vasoconstriction can redirect up to 1000 mL of fluid from the splanchnic capacitance veins into the central circulation, worsening systemic venous congestion [8]. This shift from an unstressed to a stressed volume can precipitate acute decompensated HF (ADHF) without apparent fluid weight gain. Evidence from the ESCAPE trial demonstrated that elevated right atrial pressure (RAP), rather than low CO, is the principal driver of worsening renal function in ADHF, underscoring the clinical significance of renal venous congestion in CRS [9].

### 2.2. Rising Serum Creatinine Versus Worsening Renal Function with Diuresis

Diuretics are not inherently nephrotoxic [10]. A rise in serum creatinine (SCr) with diuretics is not synonymous with worsening renal function (WRF) or acute kidney injury (AKI). Such increases often reflect a haemodynamically driven reduction in GFR from effective fluid removal, rather than renal tubular damage. Importantly, venous congestion itself can significantly elevate urea and SCr; discontinuing diuretics and administering fluids in this setting can exacerbate congestive renal dysfunction and trigger a downward spiral in CRS [11]. On the other hand, excessive diuresis can lead to intravascular volume depletion and pre-renal AKI or even acute tubular necrosis (ATN). Hence, clinical assessment for hypovolaemia, e.g., low jugular venous pressure, reduced skin turgor, or orthostatic hypotension, remains crucial. Furthermore, initiation of the renin–angiotensin–aldosterone system (RAAS) inhibitors or sodium–glucose cotransporter-2 inhibitors (SGLT2i) during hospitalisation may also cause an initial tip in eGFR by 10–20% from transient changes in glomerular haemodynamics, which are not indicative of tubular injury and may be associated with long-term benefit [12]. A >30% eGFR decline is uncommon and should trigger evaluation for other reasons, such as volume depletion or nephrotoxic exposure or intrinsic renal pathology.

Transient SCr elevation during decongestion must be carefully differentiated from true AKI, where oliguria (urine output < 0.5 mL/kg/h) persists despite high-dose diuretics [13]. While novel renal biomarkers (e.g., NGAL, KIM-1, and TIMP-2) offer promise in differentiating intrinsic renal injury from haemodynamic-induced eGFR changes, their clinical utility remains limited by availability, cost, and undefined diagnostic threshold, discussed in detail elsewhere [14]. Until further evidence emerges, urine output monitoring provides a more dynamic and responsive measure of renal function than SCr alone, especially in patients with lower muscle mass, where SCr can overestimate glomerular filtration due to reduced creatinine generation. However, the interpretation of urine output in acute HF requires clinical nuance, since renal adaptive mechanisms, e.g., antidiuresis, or paradoxical polyuria in ATN can reduce its diagnostic accuracy. B-type natriuretic peptide can serve as a complementary marker of successful decongestion [15].

Importantly, evidence from clinical trials reinforces the concept that not all rises in SCr during decongestion signify harm. The ROSE-AHF study showed that significant diuresis (median 8.4 L over 72 h) was associated with a ≥20% decline in eGFR but without evidence of tubular injury [16]. Similarly, the ESCAPE trial found that acute eGFR decline during effective decongestion was not associated with adverse outcomes in mortality or HF admissions [9]. Thus, effective decongestion takes precedence over transient SCr rises, as unresolved congestion from premature discontinuation of diuretics poses a far greater risk.

## 3. Initial Assessment and Diagnostic Workup

### 3.1. Rule out Intrinsic Renal Disease

Urine albumin–creatinine ratio (or 24 h urine in AKI) should be sent, where nephrotic levels (>220 mg/mmol) may suggest a primary glomerular pathology, e.g., diabetic or membranous nephropathy. Renal ultrasound is vital to assess for CKD (small echogenic kidneys < 8–9 cm in length with loss of corticomedullary differentiation) and to exclude obstructive uropathy. Additionally, it is crucial to exclude systemic conditions such as sepsis, cardiac amyloidosis, sarcoidosis, and Fabry disease that can simultaneously affect the heart and kidneys (type 5 CRS) [14]. Cardiac amyloidosis should be suspected when there is low QRS voltages despite left ventricular hypertrophy (LVH), autonomic dysfunction, bilateral carpal tunnel syndrome, and spinal stenosis; serum and urine electrophoresis with immunofixation with free light chains should be sent, along with a 99m Technetium DPD/PYP/HMDP bone scintigraphy to look for transthyretin amyloidosis [15,17]. Sarcoidosis should be suspected in cases of conduction abnormalities or ventricular arrhythmias, pulmonary infiltrates, uveitis, hypercalcaemia, or lymphadenopathy, which can be biopsied for non-caseating granulomas. Fabry disease, caused by α-galactosidase A enzyme deficiency leading to Gb3 accumulation, should be suspected in patients with LVH, acroparaesthesia (burning sensations of hands and feet), angiokeratoma (dark red/blue papules on lower trunk), hypohidrosis, and X-linked family history; an EDTA bottle (lavender top) should be sent for α-galactosidase A enzyme activity [6]

### 3.2. Ultrasound Assessment of Congestion

**Figure 1:**
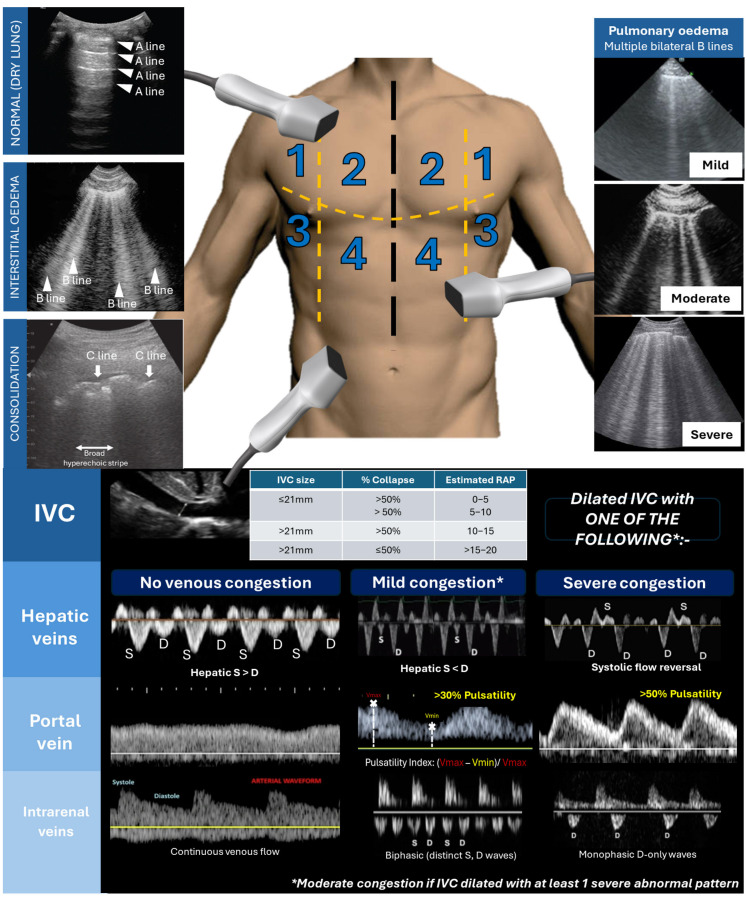
(**Top**) Thoracic ultrasound to determine the degree of pulmonary oedema (mild: 2–3 B lines; moderate: ≥4 B lines; severe: confluent B lines); C lines: short hyperechoic lines with broad hyperechoic stripe below suggest consolidation. (**Bottom**) Venous excess ultrasound based on plethoric IVC with abnormal venous waveform in at least one venous system (original figure created by author PT).

Thoracic and venous excess ultrasound (VeXUS) offers a more comprehensive volume assessment than traditional clinical examination (Figure 1). Lung ultrasound detects B-lines, which indicate increased interstitial oedema. B lines appear as discrete vertical hyperechoic reverberation artefacts extending from the pleural line to the bottom of the ultrasound screen and move synchronously with lung sliding. Three or more B-lines in two bilateral lung zones suggest pulmonary oedema with 94% sensitivity and 92% specificity [18]. VeXUS evaluates systemic venous congestion by assessing flow reversal in the intrarenal, portal, hepatic veins, and inferior vena cava (IVC). Normal portal and intra-renal venous flows are continuous, while pulsatility indicates elevated RAP. Examples of mild congestion (VeXUS 1) include a plethoric IVC (>2 cm diameter with reduced collapsibility) with biphasic intrarenal or portal vein pulsatility of 30–50%. Severe congestion (VeXUS 3) is characterised by intrarenal venous pulsatility progressing from biphasic to monophasic patterns, a portal vein pulsatility fraction > 50%, hepatic systolic flow reversal, and an IVC collapsibility index ≤ 15%. Moderate congestion (VeXUS 2) involves one of the three severe waveform patterns (Figure 1) [19]. Important caveats to VeXUS include portal hypertension in liver cirrhosis (masks venous congestion patterns), severe tricuspid regurgitation (falsely elevating VeXUS score), and those on mechanical ventilation (altering venous doppler dynamics) [19]. A high VeXUS score in CRS strongly suggests venous congestion, supporting aggressive decongestion strategies.

## 4. Decongestive Strategies in Diuretic Resistance

**Figure 2:**
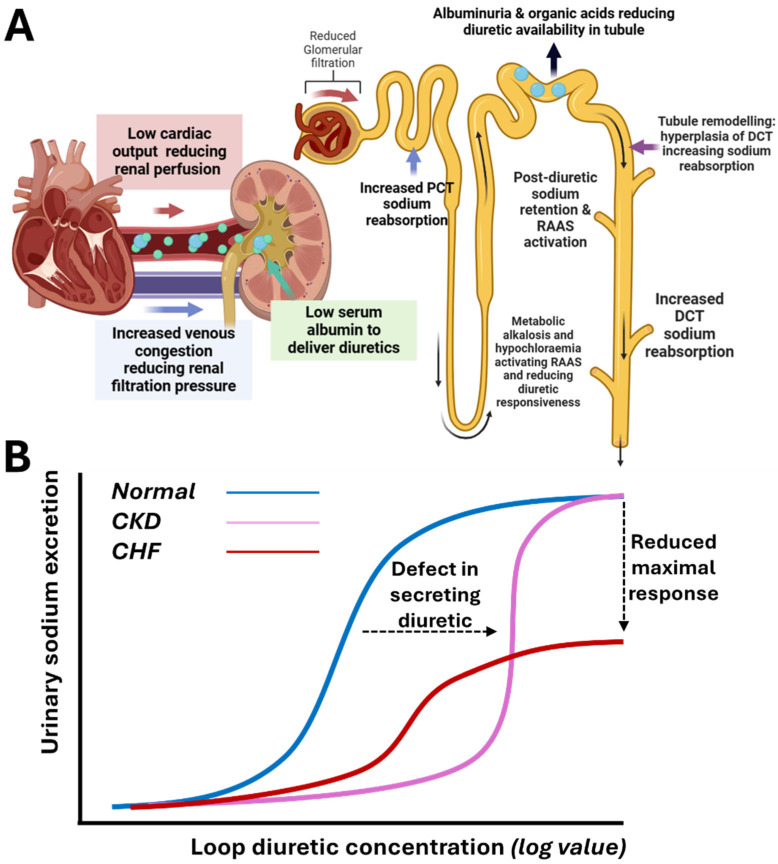
(**A**) Multifactorial causes of diuretic resistance (green dots represent loop diuretic and blue dots represent albumin forming complexes with the loop diuretic; The red arrow indicates forward perfusion and blue arrow indicates increased venous pressure. Black arrow indicates direction of natriuresis through the tubule, with dark blue arrow indicating strong factor for diuretic resistance, vs purple arrow to suggest moderate factor for diuretic resistance and blue arrow to indicate weak-moderate factor). (**B**) Dose–response curve with rightward and downward shift in heart failure (figure is original and created by author PT).

**Figure 3:**
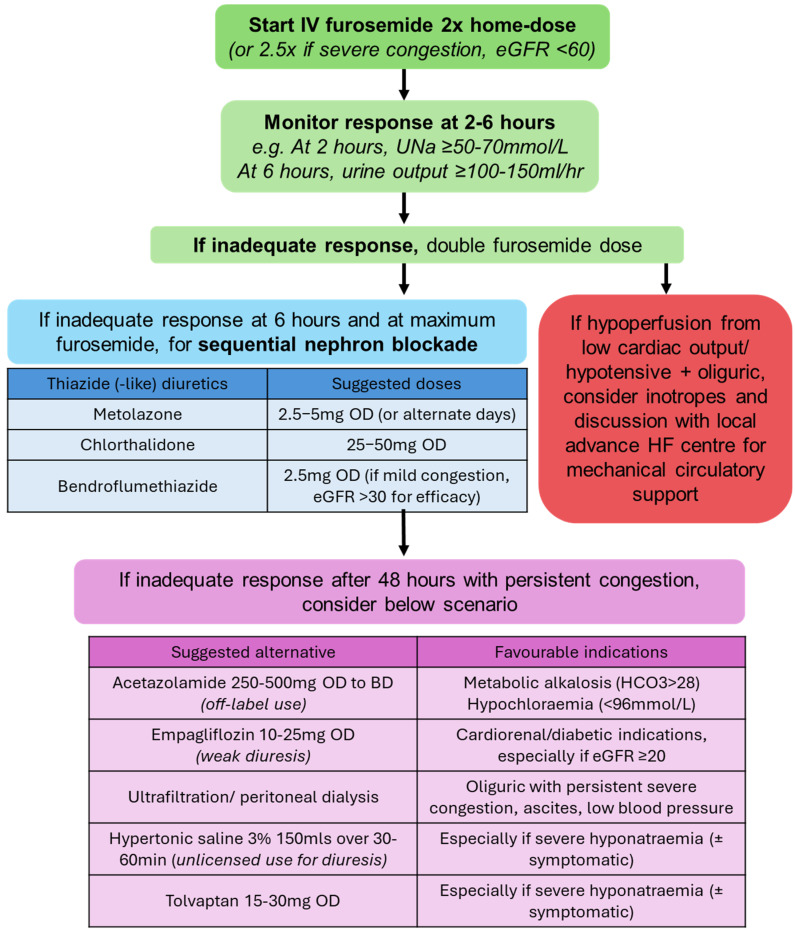
Suggested algorithm for diuretic resistance.

Diuretic resistance, defined as inadequate natriuresis and relief of volume overload despite escalating doses of intravenous diuretics, represents a critical barrier in CRS management. Common definitions include a failure to excrete ≥ 90 mmol/L urinary sodium within 72 h of intravenous 160 mg furosemide twice daily [20]. Causes include reduced renal perfusion, hypoalbuminaemia limiting diuretic delivery, underlying CKD impairing tubular secretion of diuretics, and gut oedema limiting oral absorption (Figure 2A). Moreover, chronic diuretic exposure induces distal nephron hyperplasia, enhancing sodium reabsorption and creating a pathophysiological basis for diuretic resistance [20]. These intertwined mechanisms necessitate a multifaceted approach, including sequential nephron blockade (Figure 3).

### 4.1. Furosemide Dose Titration Guided by Spot Urinary Sodium

Sodium is the main determinant of extracellular volume, making adequate natriuresis essential for effective decongestion. For diuretic naïve patients, an initial intravenous furosemide dose of 20–40 mg is recommended, adjusted based on response. For patients on chronic diuretics, the initial intravenous furosemide dose should be twice the pre-admission dose to account for the rightward and downward shift in the dose–response curve in ADHF (Figure 2B) and reduced drug delivery to the tubules in CRS. The DOSE-AHF trial showed that high-dose furosemide (2.5 × home dose) achieved greater diuresis and earlier transition to oral diuretics at 48 h, compared to low-dose therapy [21]. The feasibility and safety of natriuresis-guided diuresis were demonstrated in ENACT-HF and PUSH-AHF, irrespective of baseline eGFR [13,22]. Endorsed by the ESC HF guidelines, measuring spot urinary sodium (UNa) at 2 h provides an early indicator of natriuretic response, with satisfactory UNa being >50–70 mmol/L and/or urine output >100–150 mL/h in the first 6 h [15]. Levels below these thresholds should prompt doubling the furosemide dose or adding a second diuretic. To streamline this process, clinicians should collaborate with the laboratory team and integrate UNa results with decision-support alerts on electronic patient records.

### 4.2. Continuous Versus Bolus Furosemide

Continuous infusion offers several theoretical advantages over intermittent boluses. It maintains a steady diuretic concentration to counter the reduced tubular secretion in CKD and minimises post-diuretic sodium retention, where avid sodium reabsorption and fluid re-accumulation occurs as the furosemide effect wanes [22]. Given the short-lived effects of intravenous furosemide, typically six hours, bolus dosing risks this post-diuretic sodium retention if subsequent doses are delayed. The DOSE-AHF trial provided limited insights into the true benefit of continuous infusion, as it lacked an initial loading dose to rapidly achieve therapeutic levels above the natriuretic threshold and used suboptimal infusion rates (<10 mg/h) [19]. Diuretic resistance often requires >10–15 mg/h. This trial also excluded patients with SCr > 265.3 µmol/L (>3 mg/dL).

### 4.3. Sequential Nephron Blockade

Sequential nephron blockade is indicated when there is inadequate natriuretic response despite optimised intravenous loop diuretics, as per the 2021 ESC HF guidelines [15]. Loop diuretics block sodium reabsorption in the loop of Henle, but sodium can still be reabsorbed in the distal tubule, contributing to diuretic resistance [23]. Thiazide-like diuretics can counteract this effect. Among these are chlorthalidone (25–50 mg once daily), which has a long half-life (40–60 h) and proven safety and efficacy in CKD stage 4 [24]. Metolazone, licensed for HF under a brand name, is commonly used at 2.5–5 mg once daily or on alternate days. Due to differences in bioavailability between the unlicensed and licensed metolazone, the latter must be prescribed under its brand name as per the Medicines and Healthcare Regulatory Agency UK. The 3T trial demonstrated that metolazone achieved significantly increased natriuresis (average UNa 104 ± 16 mmol/L) in ADHF [25], while the CARESS trial found that high-dose furosemide and metolazone, at a 10 mg dose (or, equivalently, 5 mg of the licensed form), induced diuresis comparable to ultrafiltration [26]. Bendroflumethiazide is an alternative for milder cases but loses efficacy when eGFR < 30 [7], while hydrochlorothiazide is rarely used in the UK due to limited availability and potential skin cancer risk.

Acetazolamide, a carbonic anhydrase inhibitor, reduces sodium and bicarbonate reabsorption in the proximal tubule while promoting chloride retention. Correcting for metabolic alkalosis and hypochloraemia (levels checked on blood gas) with acetazolamide can be beneficial in CRS. Metabolic alkalosis diminishes the Na-K-2Cl cotransporter responsiveness to loop diuretics and enhances sodium reabsorption in the distal nephron [26]. Sensed by the macula densa, hypochloraemia (<96 mmol/L) exacerbates diuretic resistance by activating the renin–angiotensin–aldosterone system (RAAS), promoting sodium and water retention [22,23]. The DIURESIS-CHF trial showed that acetazolamide increased urinary sodium secretion by 62% and improved loop diuretic efficiency [27]. Building on this evidence, the ADVOR trial showed that adding acetazolamide 500 mg for up to 3 days (or until euvolaemia) achieved higher diuresis and shorter hospital stays, particularly in patients with serum bicarbonate > 28 mmol/L [28,29]. However, those with eGFR < 20 were excluded, and the trial did not include thiazides or sodium–glucose cotransporter-2 inhibitors (SGLT2i), limiting generalisability. In practice, acetazolamide is started at 250 mg once or twice daily and increased up to 500 mg twice daily orally, alongside vigilant monitoring for hypokalaemia and metabolic acidosis.

SGLT2i promotes osmotic diuresis and natriuresis by inhibiting proximal tubular reabsorption of glucose and sodium. In the EMPA-RESPONSE-AHF trial, empagliflozin 10 mg/day increased urine output and net fluid in ADHF at 30 days without adverse effects on blood pressure or renal function. EMPAG-HF used a higher dose of empagliflozin 25 mg/day, initiated within 12 h of admission with ADHF, and found increased cumulative urine output (additional 2.2 L) and reduced natriuretic peptides without affecting renal function. Similarly, the EMPULSE trial showed that empagliflozin improved decongestion with benefits sustained over 90 days. These agents are effective across a wide range of renal functions, including those with eGFR ≥ 20 mL/min/1.73 m^2^ (hereafter as a number only). In the DAPA-RESIST trial comparing dapagliflozin 10 mg/day with metolazone 5–10 mg/day in diuretic-resistant HF, both achieved comparable weight reduction over a 3-day treatment period (3 vs. 3.6 kg, *p* = 0.11). Although metolazone showed greater loop diuretic efficiency (weight change per 40 mg furosemide), dapagliflozin led to fewer electrolyte disturbances than metolazone [30].

Spironolactone should not be used as a sequential nephron blockade for several reasons. It is a weak diuretic that generally takes 2–3 days for its active metabolite, canrenone, to suppress aldosterone activity. This would be unsuitable in acute CRS where rapid volume removal is crucial. The ATHENA-HF trial found no significant improvement in weight loss and urine output between spironolactone 100 mg/day and 25 mg/day but increased the risk of hyperkalaemia and rising SCr [31].

### 4.4. Hypertonic Saline Solution (HSS)

Unlike its use in symptomatic SIADH, HSS in CRS aims to improve decongestion via osmotic mobilisation of fluid from the interstitial space into the intravascular space to enhance renal blood flow and reduce RAAS activation [30,31]. The chloride content also helps modulate the macula densa response to hypochloraemia, as previously discussed. A meta-analysis found that HSS with furosemide increased diuresis and shortened hospital stays better than furosemide alone. The typical regimen involves 150 mL of 1.4–4.6% (usually 3% hypertonic saline) over 30–60 min up to twice daily alongside high-dose IV furosemide [31]. HSS has been shown to be safe in ambulatory HF patients, though the trial excluded those with SCr > 221 µmol/L (>2.5 mg/dL) [32,33]. HHS should be avoided in hypernatraemia or severe pulmonary oedema. Sodium levels should be checked 1–2 h after administration, ensuring a sodium increase of 4–6 mmol/L in the first 6 h (<10 mmol/L in 24 h).

### 4.5. Vasopressin-2 Receptor Antagonist

Tolvaptan promotes aquaresis by increasing free water excretion without significant sodium loss by blocking vasopressin-2 receptors in the collecting ducts. This can rapidly correct hyponatraemia and reduce congestion without worsening renal function. The ACTIV in the CHF trial showed that tolvaptan improved dyspnoea and reduced congestion within 24–48 h of initiation, with 30 mg offering the best balance between efficacy and safety [34]. However, the 3T trial comparing tolvaptan, metolazone, and chlorothiazide found no significant difference in fluid weight loss [25]. As an off-label indication for fluid overload, particularly in those with significant hyponatraemia, the typical dosing is 15–30 mg once daily.

## 5. Renal Replacement Therapies in Diuretic Resistance

### 5.1. Peritoneal Dialysis

Peritoneal dialysis (PD) provides intracorporeal ultrafiltration, removing sodium and fluid with minimal haemodynamic compromise, ideal for patients with hypotension [35,36]. The typical regimen involves 4–6 exchanges per day using 1.5–4.25% dextrose solutions, with the 4.25% solution achieving 600–1000 mL ultrafiltration per exchange in cases of severe fluid overload. A meta-analysis of PD in refractory congestive HF found it reduced hospital stay, improved exercise capacity, and enhanced left ventricular systolic function [37]. Patient selection for PD is crucial. It is particularly beneficial for patients with significant ascites from systemic venous congestion, as relieving intra-abdominal pressure can improve renal function [38]. However, PD is contraindicated in patients with a high risk of peritonitis, poor wound healing, or abdominal wall defects, e.g., hernias or surgical scars.

### 5.2. Ultrafiltration

Mullen et al. (2023) challenged conventional sequential nephron blockade strategies, noting that adding acetazolamide, thiazides, or empagliflozin yields modest additional urine volumes (approximately 250 mL, 375 mL, and 385 mL, respectively)—inadequate for the rapid volume removal rates required in CRS [39]. In this context, ultrafiltration (UF) has re-emerged as a promising mechanical decongestion therapy in CRS, capable of removing 100–500 mL/h of isotonic fluid by pumping blood through a negatively pressured semipermeable filter system. Early evidence from the UNLOAD (2007) trial demonstrated greater weight loss and fewer 90-day re-hospitalisations than intravenous diuretics [40]. However, enthusiasm waned after the CARRESS-HF (2012) trial, which found no significant differences between UF and an aggressive individualised diuretic protocol [26]. However, this study was limited by high crossover rates and its non-physiologically fixed UF rates at 200 mL/h. Interest in UF was reignited by the AVOID-HF (2016), which showed that adjustable UF (rates up to 500 mL/h) achieved superior decongestion than diuretics, albeit with increased catheter-related complications [41,42]. Importantly, AVOID-HF excluded patients with SCr > 265.3 µmol/L (>3 mg/dL). The ongoing REVERSE-HF will clarify the role of UF in CRS using more refined protocols and modern technology (Aquadex^®^ System, Nuwellis Inc., San Diego, CA, USA). Additionally, modern miniaturised UF devices can now offer portable alternatives compatible with peripheral venous access, capable of removing around 1.8L over six hours [43].

## 6. Role of Inotropic Agents in Acute CRS

Contrary to common practice, withholding diuretics due to worsening hypotension is counterproductive in CRS with volume overload. Hypotension in this setting often signals severe CRS and possible cardiogenic shock (i.e., tissue hypoperfusion from cardiac impairment regardless of blood pressure), necessitating urgent decongestion. Selective use of inotropic agents can provide haemodynamic support for diuresis. Low-dose dopamine (<3 µg/kg/min) theoretically promotes renal vasodilatation, enhancing renal perfusion and natriuresis. The ROSE trial demonstrated that low-dose dopamine over 72 h improved urine output in patients with LVEF ≤ 50% or systolic blood pressure ≤ 114 mmHg [44]. Dobutamine, a β1-adrenergic agonist, supports patients with low cardiac output while providing mild afterload reduction via β2-mediated peripheral vasodilation. However, its limitations include tachyarrhythmias, worsening of myocardial ischaemia, and reduced efficacy in patients on chronic beta-blockers. The LIDO trial found that levosimendan, a calcium sensitiser, improved renal function in patients with type 1 CRS more effectively than dobutamine [45]. However, levosimendan remains limited in terms of UK availability and carries a risk of vasoplegia (profound hypotension) and cardiac arrhythmias.

## 7. Long-Term HF Management in CRS

It is important for clinicians to recognise the eGFR cut-offs in pivotal trials that underpin guideline-directed medical therapies (GDMTs) for HF, as these limitations influence the applicability of treatments in patients with CRS, summarised in Table 2 [15]. When using these therapies beyond the eGFR trial’s inclusion criteria, clinicians will rely on off-label use guided by clinical judgement and individual patient characteristics. In terms of device therapy, cardiac resynchronisation therapy (CRT) remains a cornerstone for eligible patients with LVEF ≤ 35%, NYHA Class II-IV (ambulatory), and QRS > 130 (ideally ≥ 150 ms, with a left bundle branch block. The MIRACLE trial found that CRT increased eGFR by +2.7 ± 1.2 at 6 months in patients with baseline eGFR < 60 [46]. This trial excluded patients with eGFR < 30. Theoretically, CRT may improve cardiac output and, in turn, renal function to allow the introduction of GDMT, which was previously not possible due to renal function. Importantly, optimal CRS management necessitates a multidisciplinary approach, bringing together cardiologists, nephrologists, pharmacists, specialist nurses, and other specialists within joint clinics, centred around a patient-centred model of care.

## 8. Future Directions in CRS Management

Ongoing clinical trials are advancing novel strategies to target the intricate interplay between renal hypoperfusion and venous congestion in CRS. The pivotal DRAIN-HF study is evaluating a circulatory support device to enhance renal perfusion in ADHF with persistent congestion (NCT05677100), offering hope for restoring renal blood flow in low CO states. For venous congestion relief, the ongoing single-arm clinical trial (NCT05759806) is evaluating a novel passive flow diversion device positioned in the IVC below the renal veins, aiming to reduce renal venous pressure. In parallel, diagnostic advances are being explored, with multiple studies examining VeXUS ultrasound for guiding diuretic therapy in CRS (e.g., NCT06786728 and NCT06065163). These studies reflect a multidimensional approach combining haemodynamic support, decongestive interventions, and image-guided diuresis to tackle CRS.

## 9. Conclusions

CRS demands a tailored multifaceted approach grounded in pathophysiological understanding and a robust evidence base. Effective management hinges on precise volume assessment through point-of-care ultrasound and targeted decongestion strategies guided by objective markers including spot urinary sodium. The cornerstone of therapy remains aggressive decongestion through strategic sequential nephron blockade, even in the face of a transient serum creatinine rise that reflects haemodynamic adaptation rather than true renal injury. Practical tools such as the diuretic resistance algorithm in Figure 3 can guide clinicians in navigating CRS management. For cases of refractory overload with reduced urine output, ultrafiltration has re-emerged as a valuable intervention, while inotropic support in selective cases remains essential for patients with low cardiac output. Beyond acute management, long-term guideline-directed medical therapy should be prescribed according to the patient’s renal function, respecting the eGFR inclusion boundaries established in trials. By adopting this nuanced approach, we can potentially improve outcomes in this challenging patient population.

## Figures and Tables

**Table 1:** Classification of cardiorenal syndrome proposed by Ronco et al. (2008) [4].

Type of CRS	1	2	3	4	5
Acute vs. chronic	**Acute**	**Chronic**	**Acute**	**Chronic**	**Secondary**
Primary dysfunction	Heart	Heart	Kidney	Kidney	Both
Secondary disease	Kidneys	Kidneys	Heart	Heart	Both
Example of causes	Acute MI ADHF cardiogenic shock	Chronic congestive cardiac failure	AKI, Acute tubular necrosis, Acute GN	Chronic glomerular disease	Sepsis, infiltration, diabetes, vasculitis, liver cirrhosis
Mechanism	Acutely low CO, suddenly high CVP, RAAS/SNS activation	Chronically low CO, high CVP, RAAS activation, endothelial dysfunction	Electrolyte imbalance, hypertension, increased preload to RV, RAAS and SNS activation	Electrolyte imbalance, anaemia, chronic inflammation RAAS/SNS activation	Oxidative stress, Cytokine Inflammation

Red columns indicate heart is the primary organ dysfunction, yellow columns indicate kidneys are the primary organ dysfunction and green column being both organs. Abbreviations: ADHF: acute decompensated heart failure; AKI: acute kidney injury; CO: cardiac output; CVP: central venous pressure; GN: glomerulonephritis; MI: myocardial infarction; RAAS: renin–angiotensin–aldosterone system; RV: right ventricle; SNS: sympathetic nervous system.

**Table 2:** eGFR cut-offs for specific guideline-directed therapies in HFrEF [21].

Drug/Intervention	Trials	eGFR Exclusion	Main Finding(s)
Beta-blockers	CIBIS-II MERIT-HF	No limit	Reduce mortality and HFH
ACE inhibitors (e.g., enalapril)	CONSENSUS SOLVD	SCr > 300 µmol/L (3.4 mg/dL) SCr > 176.5 µmol/L (2 mg/dL)	Reduce HF-related mortality and slow CKD progression
Angiotensin-receptor blockers (e.g., candesartan, valsartan)	CHARM ValHEFT	SCr > 265 µmol (3 mg/dL)	Reduce CV mortality and HFH
Sacubitril/Valsartan	PARADIGM-HF	eGFR ≤ 30	Reduced risk of CV death and HFH; slowed eGFR decline and ESRD
SGLT2 inhibitors	DAPA-CKD DAPA-HF EMPEROR-Reduced	eGFR ≤ 25 eGFR ≤ 30 eGFR ≤ 20	Slowed CKD progression Reduced CV death and HFH
Mineralocorticoid antagonists	RALES EMPHASIS-HF	SCr > 221 µmol (>2.5 mg/dL) eGFR ≤ 30	Reduce mortality and HFH
Cardiac re-synchronisation therapy	MIRACLE MADIT-CRT	eGFR < 30 in MIRACLE No cut-off in MADIT-CRT	Increased eGFR by 2.7 ± 1.2 Reduce HFH and mortality
Finerenone	FIDELIO-DKD FIGARO-DKD FINEARTS	eGFR ≤ 25 eGFR ≤ 25	Reduced risk of renal failure, eGFR decline or renal-related deaths Reduced risk of worsening HF (EF > 40%)

Abbreviations: CKD: chronic kidney disease; eGFR: estimated glomerular filtration rate (mL/min/1.73 m^2^); HFH: heart failure hospitalisation; HFrEF: heart failure with reduced ejection fraction; SCr: serum creatinine; SGLT2: sodium–glucose cotransporter-2.

## Data Availability

No new data were created or analysed in this study. Data sharing is not applicable to this article.
